# Are Pericentric Inversions Reorganizing Wedge Shell Genomes?

**DOI:** 10.3390/genes8120370

**Published:** 2017-12-07

**Authors:** Daniel García-Souto, Concepción Pérez-García, Juan J. Pasantes

**Affiliations:** Dpto. Bioquímica, Xenética e Inmunoloxía, Universidade de Vigo, E-36310 Vigo, Spain; danielgarciasouto@gmail.com (D.G.-S.); concepcionperezgar@gmail.com (C.P.-G.)

**Keywords:** *Donax*, chromosome, fluorescent in situ hybridization, histone genes, ribosomal RNA genes, GC-rich heterochromatin, pericentric inversions

## Abstract

Wedge shells belonging to the Donacidae family are the dominant bivalves in exposed beaches in almost all areas of the world. Typically, two or more sympatric species of wedge shells differentially occupy intertidal, sublittoral, and offshore coastal waters in any given locality. A molecular cytogenetic analysis of two sympatric and closely related wedge shell species, *Donax trunculus* and *Donax vittatus*, was performed. Results showed that the karyotypes of these two species were both strikingly different and closely alike; whilst metacentric and submetacentric chromosome pairs were the main components of the karyotype of *D. trunculus*, 10–11 of the 19 chromosome pairs were telocentric in *D. vittatus*, most likely as a result of different pericentric inversions. GC-rich heterochromatic bands were present in both species. Furthermore, they showed coincidental 45S ribosomal RNA (rRNA), 5S rRNA and H3 histone gene clusters at conserved chromosomal locations, although *D. trunculus* had an additional 45S rDNA cluster. Intraspecific pericentric inversions were also detected in both *D. trunculus* and *D. vittatus*. The close genetic similarity of these two species together with the high degree of conservation of the 45S rRNA, 5S rRNA and H3 histone gene clusters, and GC-rich heterochromatic bands indicate that pericentric inversions contribute to the karyotype divergence in wedge shells.

## 1. Introduction

Genomic data obtained in the last two decades have revealed that chromosomes display a plethora of reorganizations [1,2]. The patterns of chromosomal rearrangements are, in many cases, lineage-specific [3]. Among those rearrangements, chiefly chromosomal inversions can confer varying degrees of fitness in different habitats and intensify reproductive isolation [1,4,5], as mating between individuals with different karyotypes will produce descendants heterozygous for chromosomal rearrangements that will have reduced fertility and/or recombination reduced within the rearranged chromosomal regions.

The wedge shells of the family Donacidae (Bivalvia, Heterodonta) are the dominant bivalves in exposed beaches from tropical and temperate coastal waters [6]. The systematic classification of this family has not yet been fully resolved [7]. Among the unsolved problems are the assignation of 88 of the 107 species to the genus *Donax* [7] and the lack of agreement on the taxonomic status of many of its taxa [8]. Although some wedge shell specimens are easily identifiable according to shell morphology, others can only be recognized after studying shell characteristics, which exhibit a great variability, and hence intermediate states between taxa are often observed [9,10]. This is further complicated by the presence of two or more geographically-sympatric species of *Donax* differentially occupying intertidal, sublittoral, and offshore regions in almost any given locality [6].

Although different kinds of genetic methodologies have been used for addressing taxonomical problems [8,11,12,13,14,15,16] and characterizing repetitive DNA sequences [17,18,19,20,21,22] in wedge shells, chromosome studies are neglected. Except for the determination of a diploid chromosome number of 2n = 38 in *Donax variabilis* [23], karyological data on the species of the family Donacidae are restricted to the abrupt wedge shell *Donax trunculus* [19,21,24,25]. The karyotype of *Donax trunculus* (2n = 38) is entirely [21,24] or almost entirely [25] composed of bi-armed chromosome pairs, many of which bear GC-rich heterochromatic bands [21,25]. In this wedge shell species, the chromosomal locations of major rDNAs [21,25], telomeric sequences [19,21], and a GC-rich satellite DNA [21] are also known.

As part of our ongoing bivalve cytogenetic research program, we found that in another species of wedge shell, *Donax vittatus*, the metaphase plates contained a high proportion of chromosome pairs with hardly detectable short arms, i.e., telocentric chromosomes. *D. trunculus* and *D. vittatus* are closely related taxa, whose status as separated species is still under debate [7]. These two species live in sympatry in the exposed beaches of northwest Spain, although they occupy intertidal and sublittoral habitats, respectively. Since these features make them excellent candidates for gaining knowledge on the chromosome diversification patterns in bivalves, we collected *D. trunculus* and *D. vittatus* specimens from sympatric and allopatric populations and analyzed their chromosomes by means of 4′,6-diamidino-2-phenylindole (DAPI)/chromomycin A3 (CMA) and DAPI/propidium iodide (PI) fluorescence staining and fluorescent in situ hybridization (FISH) with 28S rDNA, 5S rDNA, core histone gene, and telomeric probes.

## 2. Materials and Methods

### 2.1. Biological Material

**Figure 1:**
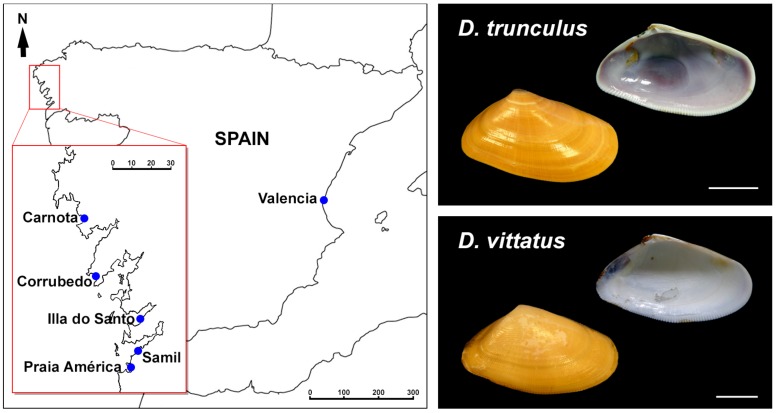
Collection localities and pictures of representative *Donax trunculus* and *Donax vittatus* analyzed.

Wedge shells specimens were collected (Figure 1) from exposed beaches in Galicia (northwest Spain). The specimens were identified as the abrupt wedge shell *Donax trunculus* Linnaeus, 1758, or the banded wedge shell *Donax vittatus* (da Costa, 1778) according to their shell characteristics. Taking into account the observed differences in the karyotype composition between the Mediterranean [24] and Atlantic [25] populations of *D. trunculus*, we also included specimens of the abrupt wedge shell collected in the Gulf of Valencia (east Spain) in our study. In all cases, wedge shells were transported to the laboratory, maintained in tanks of 5 L filtered seawater at 18 ± 1 °C and fed on microalgae to promote somatic growth [26,27].

### 2.2. Mitotic Chromosome Preparation and Fluorochrome Staining

Chromosome preparations were obtained following the technique described by Martínez-Expósito et al. [28,29]. Wedge shell specimens were housed in 0.5-L beakers and exposed to colchicine (0.005%) for 12 h. Gill and mantle tissues were then excised and immersed in 50% (20 min) and 25% (20 min) seawater and fixed with ethanol/acetic acid for 1 h. Chromosome spreads were obtained by dissociating small pieces of tissue in 60% acetic acid and dropping the cellular suspension onto clean slides heated to 50 °C.

Fluorochrome staining was performed as described by Pérez-García et al. [30]. After controlling the quality of the chromosome preparations by phase contrast microscopy (Nikon, Tokyo, Japan), selected slides were stained with CMA (0.25 mg/mL) for 2 h, counterstained with 4′,6-diamidino-2-phenylindole (DAPI; 0.14 μg/mL) for 8 min, washed in tap water, air-dried, and mounted with antifade (Vectashield, Vector, Burlingame, CA, USA). Slide visualization and photography were performed using a Nikon Eclipse-800 microscope equipped with an epifluorescence system. Separated images for each fluorochrome were obtained with a DS-Qi1Mc charge coupled device (CCD) (Nikon, Tokyo, Japan) controlled by the NIS-Elements v. 3.00 software (Nikon). Merging of the images was performed with Adobe Photoshop CS2 (San Jose, CA, USA). Following visualization and photography, chromosome preparations were re-stained with a combination of DAPI and PI (0.07 μg/mL), washed in tap water, air-dried, mounted in antifade, and photographed again.

### 2.3. DNA Extraction, PCR Amplification and DNA Sequencing

DNA extraction was performed with the E.Z.N.A. Mollusc DNA Kit (Omega, Norcross, GA, USA) following the protocol provided by the manufacturer. The purity and the concentration of the genomic DNA samples were assessed employing a BioDrop μLITE (Biodrop, Cambridge, UK).

DNA sequences were amplified in a GeneAmp PCR system 9700 (Applied Biosystems, Foster City, CA, USA) in 50-μL solutions containing 125 ng of genomic DNA, 50 μM each dNTP, 50 μM of each primer, 1× PCR buffer, 15 μM of MgCl_2_, and 5 U of JumpStart™ Taq DNA Polymerase (Sigma, St. Louis, MO, USA). Amplifications included an initial denaturation step at 95 °C (2 min), 35 amplification cycles (Table 1) and a final extension at 72 °C (5 min). PCR products were examined by electrophoresis on 2% agarose gels.

The amplified mitochondrial cytochrome oxidase I COI gene was purified (FavorPrep^TM^ GEL/PCR Purification Kit, Favorgen, Ping-tung, Taiwan) and sequenced (Centro de Apoio Científico-Tecnolóxico á Investigación (CACTI), University of Vigo, Vigo, Spain) in both directions in an ABI PRISM 3730 Genetic Analyzer (Applied Biosystems) using a BigDye Terminator v3.1 Cycle Sequencing Kit (Applied Biosystems). The sequences were edited with BioEdit v. 7.1.11 [31] and aligned with MUltiple Sequence Comparison by Log- Expectation (MUSCLE) set to default parameters using MEGA7 [32]. Sequence similarity searches were performed using the Basic Local Alignment Search Tool algorithm (BLAST), available at the National Center for Biotechnology Information (NCBI, http://www.ncbi.nlm.nih.gov/blast). The MegaBLAST algorithm set to default parameters was employed against the NCBI nucleotide collection (http://www.ncbi.nlm.nih.gov/nucleotide/) database. Relationships among sequences were inferred from a maximum likelihood (ML) tree using a HKY + I substitution model. ML reliability was assessed with 500 bootstrap replicates. Analyses were performed on MEGA7 [32].

As the analysis of the restriction fragment length polymorphism (RFLP) of the COI gene PCR products has been proposed as a tool to identify the European species of *Donax* [14], we also compared the expected patterns of fragment sizes generated by *Alu*I, *Hae*III, and *Msp*I on our sequences with those obtained by Nantón et al. [14].

### 2.4. Fluorescent in Situ Hybridization (FISH)

Metaphase chromosome spreads obtained from wedge shells collected at all localities were single, double and sequentially hybridized using 5S and 28S rDNA and H3 histone gene probes [27,33,34,35]. 2The 8S rDNA probes were labeled with biotin-16-dUTP (Roche Applied Science) and/or digoxigenin-11-dUTP (10× DIG Labeling Mix, Roche Applied Science) using a nick translation kit (Roche Applied Science). H3 histone gene and 5S rDNA probes were directly labeled by PCR either with biotin-16-dUTP (20 μM) or digoxigenin-11-dUTP (5 μM).

Chromosome preparations were digested with RNase and pepsin before denaturation (70 °C, 2 min) and hybridized overnight at 37 °C. Biotin was detected with fluorescein isothiocyanate (FITC)-conjugated avidin and biotinylated anti-avidin (Vector) whereas digoxigenin was detected with anti-digoxigenin antibodies conjugated with tetramethylrhodamine isothiocyanate (TRITC; Sigma). Chromosome preparations were counterstained with DAPI, mounted with antifade, and examined by fluorescence microscopy [27,33]. Separated images for each fluorochrome were recorded, pseudo-colored, and merged as indicated above. In addition, we also performed FISH with a telomeric (C_3_TA_2_)_3_ peptide nucleic acid (PNA) probe (Applied Biosystems) following the protocol indicated by the supplier.

Chromosome counting and karyotype analysis were performed in 40 specimens, 20 per species (10 females, 10 males). For each species, 20 complete metaphase plates showing FISH signals were used to construct karyotypes. Chromosome and arm lengths were carefully measured and relative lengths and centromeric indices calculated.

## 3. Results

All partial COI gene sequences obtained in this work were independently compared using BLAST with those stored in GenBank database. The nucleotide sequences for all wedge shell specimens morphologically assigned to *D. trunculus* (GenBank accession numbers KY951431 to KY951446) displayed a high degree of similarity (>98%) with the *D. trunculus* sequences stored in GenBank (accession numbers KC429143 and KY780364). Concordantly, all our *D. vittatus* sequences (GenBank accession numbers KY951416 to KY951430) also displayed similarities higher than 98% with all *D. vittatus* GenBank sequences (accession numbers KR084728, KR084687, KR084687, KR084728, MF668318 to MF668376, KY780366). The resulting phylogenetic tree derived from the mitochondrial COI gene dataset obtained in this work is shown in Figure S1. Moreover, the positions occupied by the restriction targets for *Alu*I, *Hae*III, and *Msp*I on the obtained COI gene sequences would generate RFLP patterns in line with those predicted by Nantón et al. [14] for *D. trunculus* and *D. vittatus* (Figure S2).

**Figure 2:**
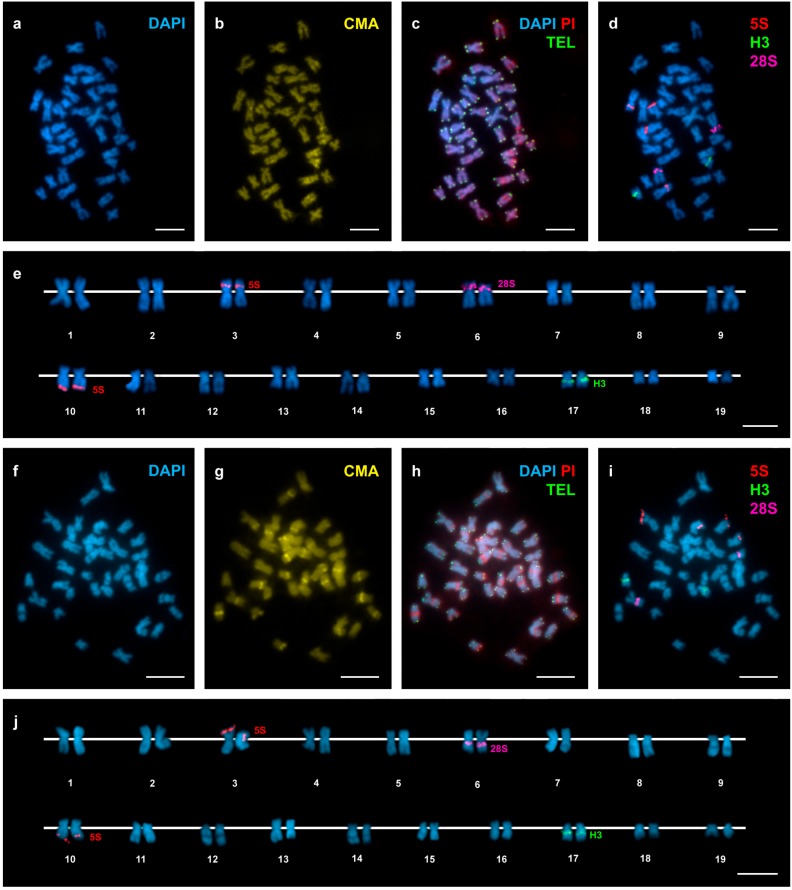
Mapping of telomeric sequence, 5S rDNA, 28S rDNA, and H3 histone gene clusters on *Donax trunculus* chromosomes. Sequential fluorochrome staining of mitotic metaphase plates shows 4′,6-diamidino-2-phenylindole (DAPI)-negative regions (**a**,**f**) that fluoresce yellow after chromomycin A3 (CMA) (**b**,**g**) and bright red after DAPI/ propidium iodide (PI) (**c**,**h**) staining in both Atlantic (**a**–**c**) and Mediterranean (**f**–**h**) specimens of *Donax trunculus*. Hybridization of the same metaphase plates (**c**,**h**) with a telomeric peptide nucleic acid PNA probe discloses signals (TEL, green) at both ends of every chromosome. Sequential fluorescent in situ hybridization (FISH) experiments using major and minor rDNA and H3 histone gene probes on the same metaphase plates (**d**,**i**), and the corresponding karyotypes (**e**,**j**), show H3 histone gene signals (H3, green) intercalated in the long arms of chromosome pair 17. Minor rDNA probes (5S, red) map to two locations, intercalated in the short arms of chromosome pair 3 and subterminal to the long arms of chromosome pair 10. Major rDNA signals (28S, magenta) are intercalated in the short arms of subtelocentric chromosome pair 6 in Atlantic specimens (**d**,**e**) and subcentromeric to the long arms of metacentric pair 6 in Mediterranean specimens (**i**,**j**). Note that none of the chromosome pairs is telocentric. Scale bars, 5 μm.

**Figure 3:**
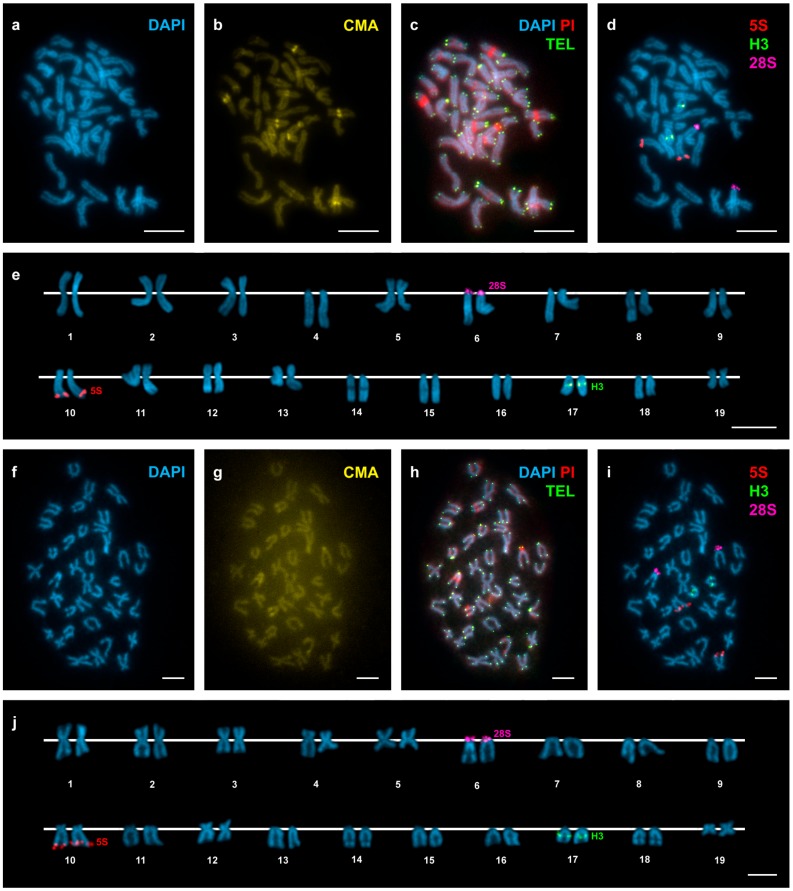
Mapping of telomeric sequence, 5S rDNA, 28S rDNA, and H3 histone gene clusters on *Donax vittatus* chromosomes. Sequential fluorochrome staining of mitotic metaphase plates shows DAPI-negative regions (**a**,**f**) that fluoresce yellow after CMA (**b**,**g**) and bright red after DAPI/PI (**c**, **h**) staining in *Donax vittatus* specimens from both Samil (**a**–**c**) and all the other Galician (**f**–**h**) populations. Hybridization of the same metaphase plates (**c**,**h**) with a telomeric PNA probe discloses signals (TEL, green) at both ends of every chromosome. Sequential FISH experiments using major and minor rDNA and H3 histone gene probes on the same metaphase plates (**d**,**i**), and the corresponding karyotypes (**e**,**j**), show H3 histone gene signals (H3, green) intercalated in the long arms of telocentric chromosome pair 17. Minor rDNA signals (5S, red) are subterminal to the long arms of subtelocentric chromosome pair 10. Major rDNA signals (28S, magenta) overlap the short arms of telocentric chromosome pair 6. Note the presence of 10 telocentric chromosome pairs in specimens from Samil (4, 6, 7, 8, 9, 14, 15, 16, 17 and 18) and 11 in those from the remaining Galician populations (6, 7, 8, 9, 11, 13, 14, 15, 16, 17 and 18). Scale bars, 5 μm.

In agreement with previous reports [21,24,25], *D. trunculus* showed a diploid chromosome number of 2n = 38. The chromosome complement of 2n = 38 for *D. vittatus* is described here for the first time. Representative metaphase plates and karyotypes (presenting chromosome pairs in decreasing order of size) of these species appear in Figure 2 and Figure 3. The karyotype of *D. trunculus* (Figure 2e,j) is composed of bi-armed (metacentric, submetacentric, and subtelocentric) chromosome pairs, whereas 10 or 11 of the 19 chromosome pairs in *D. vittatus* are undoubtedly telocentric (Figure 3e,j). Both wedge shells exhibit DAPI-negative (Figure 2a,e,f,j and Figure 3a,e,f,j), CMA-positive (Figure 2b,g and Figure 3b,g), PI-positive (Figure 2c,h and Figure 3c,h), GC-rich heterochromatic bands in a number of chromosome pairs: 14 in *D. trunculus* and 6 in *D. vittatus*. Telomeric sequence signals were restricted to the terminal regions of the chromosomes in both *D. trunculus* (Figure 2c,h) and *D. vittatus* (Figure 3c,h), with no evidence of signals in any other chromosomal regions.

**Figure 4:**
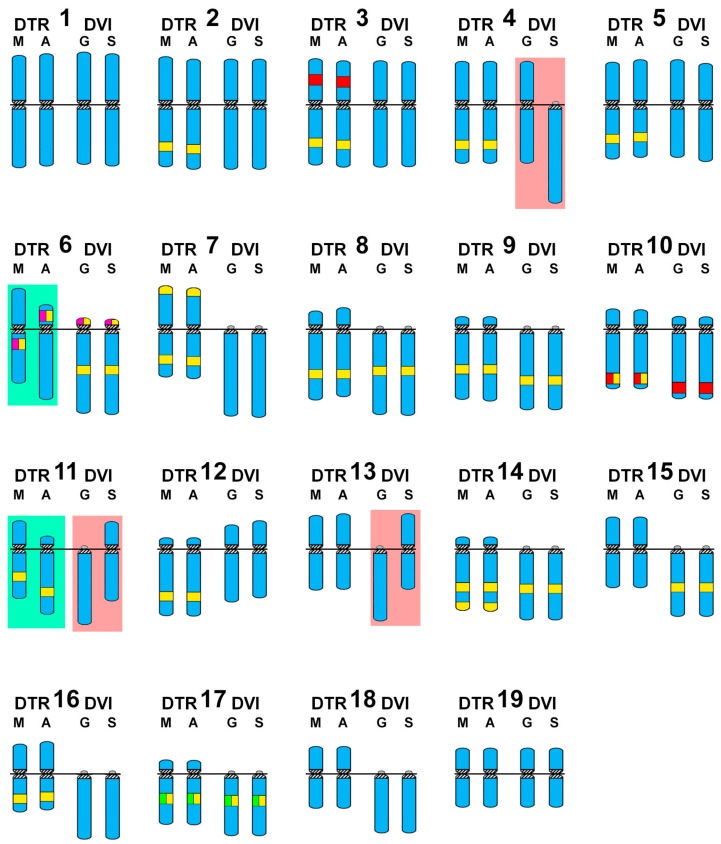
Schematic representation of the wedge shell haploid chromosome complements. For comparative purposes, the schemas of chromosomes 1 to 19 in *Donax trunculus* (DTR) Mediterranean (M) and Atlantic (A) populations and *Donax vittatus* (DVI) Galician (G) and Samil (S) populations are represented in quartets. DAPI−/CMA+ bands (yellow), 5S rDNA (red), 45S rDNA (magenta) and H3 histone gene (green) are also represented. The chromosomes showing the most remarkable intraspecific differences are shadowed in light green for *Donax trunculus* and light pink for *Donax vittatus*.

Single, double, and sequential hybridization experiments were performed to locate the H3 histone, 28S rRNA, and 5S rRNA gene clusters on the chromosomes of the two species. The clusters of H3 histone genes mapped to intercalary positions in 17q. The chromosome was submeta/subtelocentric in *D. trunculus* (Figure 2d,e,i,j) and telocentric in *D. vittatus* (Figure 3d,e,i,j). In accordance with previous reports [21,25,27], major rDNAs map to an intercalary location in 6p (subtelocentric) in all Atlantic specimens of *D. trunculus* (Figure 2d,e) but it is subcentromeric to 6q (metacentric) in all Mediterranean *D. trunculus* specimens (Figure 2i,j). In *D. vittatus*, major rDNAs also map to 6p but this chromosome is telocentric (Figure 3d,e,i,j). *D. trunculus* and *D. vittatus* presented different numbers of 5S rDNA clusters. Both wedge shell species displayed subterminal signals on 10q, but this chromosome was submetacentric in *D. trunculus* and subtelocentric in *D. vittatus* (Figure 2d,e,i,j and Figure 3d,e,i,j). *D. trunculus* also presented another signal intercalary to 3p (Figure 2d,e,i,j). A schematic, comparative representation integrating all cytogenetic results is shown in Figure 4.

Atlantic and Mediterranean *D. trunculus* karyotypes were very different. Chromosome pair 6, which is subtelocentric in the Atlantic specimens, is clearly metacentric in Mediterranean abrupt wedge shells. Chromosome pair 11 also differs in morphology, as it is subtelocentric in Atlantic specimens but submetacentric in the Mediterranean ones (Figure 2 and Figure 4). A similar situation was detected for three chromosome pairs in *D. vittatus*; chromosome pair 4 is telocentric and pairs 11 and 13 are metacentric in the Samil specimens. In all other banded wedge shells chromosome pair 4 is metacentric while pairs 11 and 13 are telocentric (Figure 3 and Figure 4).

Interspecific karyotype comparison (Figure 4) clearly shows that the telocentric chromosome pair numbers 6, 7, 8, 9, 11, 13, 14, 15, 16, 17, and 18 found in the majority of *D. vittatus* specimens are bi-armed pairs, being meta and submetacentric (7, 8, 13, 15, 16, 18) or subtelocentric (6, 9, 11, 14, 17), in Atlantic *D. trunculus*.

## 4. Discussion

The 2n = 38 diploid chromosome numbers of the two wedge shell species studied here coincide with previous reports for *Donax variabilis* [23] and *D. trunculus* [21,24,25]. This is also the usual diploid number found in most bivalve species belonging to the subclass Heterodonta [27,34,35,36,37,38,39,40,41,42,43,44].

In contrast, the karyotypes of the wedge shells studied here present some unusual characteristics for the subclass Heterodonta. Typically, those bivalve karyotypes are poor in telocentric chromosomes [41,44] and the presence of undetectable short arms in the telocentric pairs is uncommon. The best known examples are the dwarf surf clam *Mulinia lateralis* [45], three razor shell species [41,46], and one tellin shell species [44]. This is also the case for *D. vittatus* karyotypes in which, of a total of 10 or 11 telocentric chromosome pairs, only the 45S rDNA-bearing chromosome pair 6 clearly presented detectable short arms. In contrast, all the chromosome pairs in *D. trunculus* exhibited unmistakably visible short arms. These results are in agreement with previous studies on *D. trunculus* showing no [21,24] telocentric chromosome pair, or one [25].

**Figure 5:**
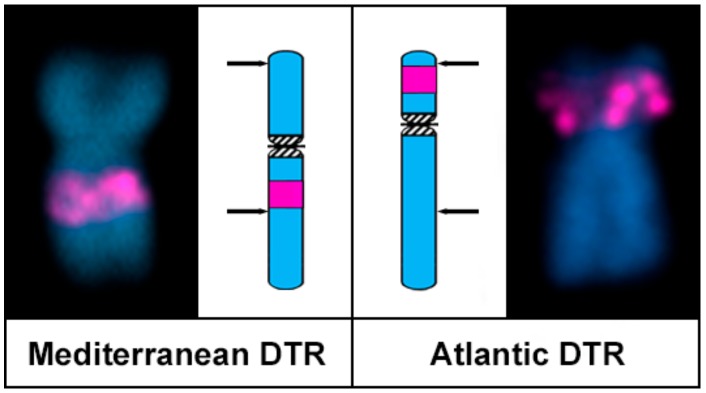
Ideogrammatic representation of the pericentric inversion differentiating chromosome 6 in Mediterranean and Atlantic populations of *Donax trunculus*. Arrows point to inversion breakpoints.

When comparing karyotypes proposed by different researchers for the same or different populations of any given bivalve species, some degree of divergence is usually detected (i.e., see the diversity in karyotype formulas given in the classical revisions by Thiriot–Quiévreux [37,38]); however, those are usually a consequence of divergent methodologies for chromosome measurement and different degrees of condensation. Nevertheless, this is not the case for the high intraspecific karyotypic diversity found in the populations of *D. trunculus* and *D. vittatus*. For instance, in accordance with previous reports [21,25], Atlantic *D. trunculus* presents a single 45S rDNA cluster on the short arms of a subtelocentric chromosome pair; in contrast, in the Mediterranean specimens of the same species the signals for the 45S rDNA cluster are subcentromeric to the long arms of a metacentric chromosome pair. Although this intrachromosomal centromere shift could be attributed to the formation of a neocentromere accompanied by a decay of the old centromere as proposed in primates [47], these phenomena are much less frequent than intrachromosomal inversions [2,48] and are not accompanied by any shifting of the other chromosomal markers [48]. As this is not the case in chromosome pair 6 of the abrupt wedge shell, the simplest explanation for the observed morphological differences in this chromosome pair in *D. trunculus* is that a pericentric inversion could have either transformed a subtelocentric pair into a metacentric one, or the other way around. In any case, one of the inversion breakpoints had to be distal, but not far away, from the 45S rDNA cluster in a chromosome arm, whilst the other would be far away from the centromere in the other arm (Figure 5). A single pericentric inversion with breakpoints close to the centromere on the short arms and proximal from the heterochromatic band on the long arms can also explain the differences observed in the chromosome pair 11. Similarly, pericentric inversions can also parsimoniously explain the interpopulational differences in chromosome morphologies found in *D. vittatus* pairs 4, 11, and 13. In any case, further developments in molecular cytogenetic technologies, i.e., obtaining suitable microdissection probes, for these bivalve species are needed to confirm that the involved rearrangements are indeed pericentric inversions.

The presence of intercalary heterochromatic GC-rich bands in bivalves is quite rare and only has been detected in three other species, the zebra mussel *Dreissena polymorpha* [49,50] and the trough shells *Spisula subtruncata* and *Mactra stultorum* [35,51]. These bands are mainly constituted of a highly amplified satellite DNA in *S. subtruncata* but this satellite was low copy in the congeneric *M. stultorum* [51]. A similar situation was expected in the two wedge shell species, that is, differentially amplified satellite DNAs as the major constituents of these heterochromatic GC-rich bands. Further work including next-generation sequencing, satellite DNA searching, and FISH-mapping [52,53,54] will be needed to confirm this.

The close genetic proximity of these two wedge shell species [7] together with the high degree of conservation of the 45S rDNA, 5S rDNA, and H3 histone gene clusters and the maintenance of the GC-rich heterochromatic bands in these taxa indicates that pericentric inversions are the main evolutionary force contributing to the karyotype divergence in wedge shells. Following this, pericentric inversions also explain the differences in chromosome shape of *D. trunculus* and *D. vittatus*.

Pericentric inversions, as well as other chromosome reorganizations, may be of considerable importance in parapatric or partly sympatric speciation processes [4], by facilitating genetic differentiation [5,55]. Although it is still not possible to confirm if chromosome rearrangements have any functional significance [56], it is clear that pericentric inversions are widespread and that, in many eukaryotes, the rearrangement breakpoints are strongly associated with repetitive sequences [3,57,58], probably as a consequence of the use of ectopic homologous sequences as a template for recombination repair [48]. Whether the relative abundance of repetitive sequences in wedge shells [17,18,20,21,22] is related to the high amount of pericentric inversions will need to be investigated further. In this sense, knowing the karyotypes of the other two species of European *Donax*, *D. semistriatus*, and *D. variegatus* [14,15,16] and, in a broader sense, performing comparative cytogenetic analyses of other bivalve groups also characterized by presenting some species rich in telocentric chromosomes and others lacking them, such as surf clams [44] and razor shells [40,45] could shed some more light on the question.

## Figures and Tables

**Table 1:** Parameters used in the PCR.

Sequence	Denaturation	Annealing	Elongation
COI gene	95 °C, 30 s	55 °C, 20 s	72 °C, 20 s
28S rDNA	95 °C, 20 s	48 °C, 20 s	72 °C, 15 s
5S rDNA	95 °C, 30 s	48 °C, 30 s	72 °C, 30 s
*h3*	95 °C, 15 s	48 °C, 15 s	72 °C, 15 s

## Supplementary Materials

The following are available online at www.mdpi.com/2073-4425/8/12/370/s1. Figure S1: Maximum likelihood tree based on mitochondrial cytochrome oxidase I COI gene sequences of species of *Donax* using the Atlantic surf clam *Spisula solidissima* as an outgroup; Figure S2: Compatibility between the expected restriction fragment patterns for *Donax trunculus* and *Donax vittatus* mitochondrial cytochrome oxidase I COI gene sequences obtained in this work and those described by Nanton et al. [14].
