# Supplementary material for: Are Pericentric Inversions Reorganizing Wedge Shell Genomes?
Source: Genes (Basel). 2017 Dec 7;8(12):370. doi: 10.3390/genes8120370 (PMC5748688; doi:10.3390/genes8120370)
Supplement: Supplementary file 1 [file genes-08-00370-s001.pdf]

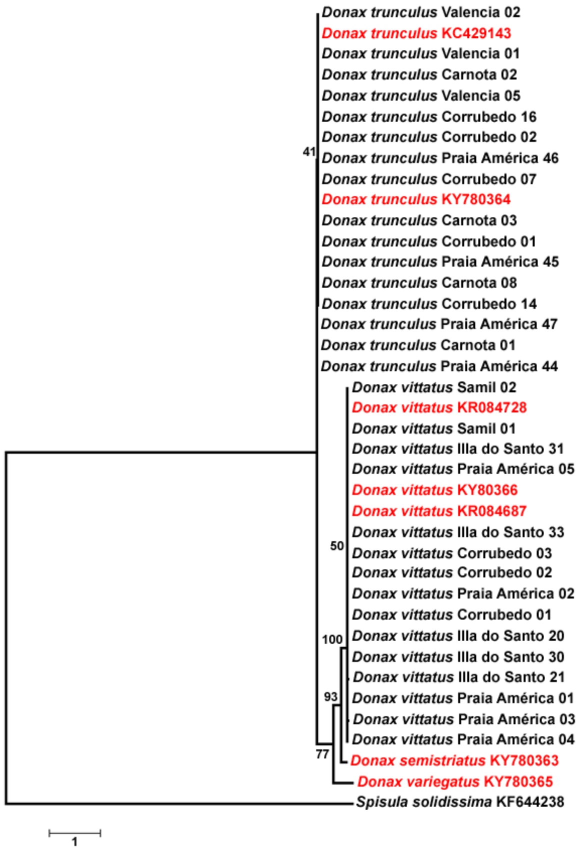

Figure S1. Maximum likelihood tree based on mitochondrial cytochrome oxidase I COI gene sequences of species of *Donax* using the Atlantic surf clam *Spisula solidissima* as an outgroup. Numbers in internal nodes, reported as percentages, indicate maximum-likelihood bootstrap support values (500 replicates).

Donax trunculus (Praia América 44)

```
ggtcaacaaatcataaagatattggaagtctttatttcgttttaggtttatgatccggccttgtggggctggtctatagagtaataatgcggactgagtta
atacatccgggcgccttttatggagagtctgtgtataatgtgttggtcacttcgcatgggttactaatgattttcttcatagtaatacctctaataattgg
gtttttggtaattgggctgttcccttactgctagcagcccctgatatagtttttgcgcggctgaacaatctaaggttctgattacttccggccgcaactg
tcctcctacttatgtctaacgaagtggaagagggtgttgggactgggtgaactctttaccctccgctatcggcttggcttggacaccccgcgcccgcgatg
gaattcatgattttagggcttcatattgcaggattatcatctatttttgctagtattaattttgtaactactggcgctaacatacgcccggagggagttgc
cccccagcgtactactttgttcgtagtatccgttgttattacgtctttcctgttggtggctgctatgcctgttttagctgcaggcttaactatactgttaa
cagatcgaaattttaacacttcttttttcgaccctgtaggggaggagatccggtgttactcattcacctattctgattttttggtcaccctgaagttta
```

Expected patterns:	582/124	58/236/412	56/53/183/201/165/48	706 bp
Nantón et al. [14]:	505/91	217/379	31/183/201/166/15	596 bp

Donax vittatus (Praia América 01)

```
ggtcaacaaatcataaagatattggtagtctttattttgttcttggactatgatctggccttgttgggctggtctacagagttataatacgtactgaacta
atacatcctggtgccttttatggtgagtctgtatataatgttttagttacgtcacatggcctacttataatttttttttatagttataccactaataattgg
ttttttggtaattgagctgtgcccctcctattagcggccccggatatggttttcgcccgtcttaataatcttagattttggttattaccagctgctacag
ttcttctacttatatcaaatgaagttgaggaaggagtaggaactggttggactttatatcctcctctttctgcttgattaggacaccctgcccctgctata
gagtttataattttagggcttcatattgctggcctatcttctatttttgctagtattaattttgttacaacaggggctaatatgcggcctgagggagtggc
ccctcagcgaactacccttttgtagtgtctgtagtaattacttcatttcttctcgtagctgctataccagttctagctgctggactgacgatacttctaa
cagatcgtaactttaatacttcctttttttgacccagtaggggtggagaccccgtattatttattcacctcttttgattttttggtcaccctgaagttta
```

Expected patterns:	219/75/270/18/124	58/102/80/196/55/13/202	244/462	706 bp
Nantón et al. [14]:	142/75/270/18/91	83/80/196/55/13/169	166/430	596 bp

Figure S2. Compatibility between the expected restriction fragment patterns for *Donax trunculus* and *Donax vittatus* mitochondrial cytochrome oxidase I COI gene sequences obtained in this work and those described by Nanton et al. [14]. The sequences display both the primers used in this work (red) and those used in [14] (bold, underlined) and the restriction targets for *Alu*I (yellow), *Hae*III (green) and *Msp*I (blue). The combination of restriction fragment sizes expected from our 706 bp sequences and those described in [14] (596-bp sequences) for each restriction enzyme is displayed in the corresponding colors.
